# Rhythms of relief: perspectives on neurocognitive mechanisms of music interventions in ADHD

**DOI:** 10.3389/fpsyg.2025.1476928

**Published:** 2025-03-03

**Authors:** Zhihui Luo, Da-Wei Zhang

**Affiliations:** ^1^School of Educational Sciences, Yangzhou University, Yangzhou, China; ^2^Department of Psychology, Jeffrey Cheah School of Medicine and Health Sciences, Monash University Malaysia, Bandar Sunway, Malaysia

**Keywords:** ADHD, music intervention, neurocognitive mechanism, executive function, brain activity, social bonding

## Abstract

Attention-deficit/hyperactivity disorder (ADHD) is a prevalent neurodevelopmental disorder characterized by multiple neurocognitive deficits. Research suggests that music interventions, both active and passive, may be an effective complementary method of addressing ADHD challenges. This narrative review discusses seven potential neurocognitive mechanisms through which music interventions may help mitigate or alleviate ADHD symptoms, including executive function enhancement, timing improvement, arousal regulation, default mode network modulation, neural entrainment, affective management, and social bonding facilitation. Our study synthesized evidence from ADHD-specific studies and examined parallels to other populations to identify possible pathways through which music therapy could exert its effect. The paper also discusses the implications of individualized music interventions tailored to specific neurocognitive profiles in ADHD, advocating additional research to refine and optimize these approaches. Overall, music therapy has substantial potential as a complementary treatment for ADHD, offering new avenues for addressing the psychosocial and cognitive aspects of this condition.

## 1 Introduction

Music engages various aspects of brain activity. As an analogy of brain activity to music composition with music as a conductor, the conductor can adjust the pitch (i.e., neurotransmitters; Chanda and Levitin, 2013), shape the melody (i.e., brain regional activation; Tramo, 2001), and fine-tune the harmony (i.e., brain interregional coupling; Alluri et al., 2012). With this impact, ‘music as medicine’ is increasingly recognized as a therapeutic tool for neurological and psychiatric conditions (Sihvonen et al., 2017; Yinger and Gooding, 2014).

Recently, this therapeutic potential has been applied to attention-deficit/hyperactivity disorder (ADHD). ADHD is a neurodevelopmental condition characterized by core symptoms of inattention, hyperactivity, and impulsivity [American Psychiatric Association (APA), 2013]. It affects approximately 5% of youths worldwide, with a roughly two-to-one male-to-female ratio, and persists into adulthood in about two-thirds of cases (Faraone et al., 2021). The condition often impairs academic, social, and occupational functioning and has high comorbidity with other psychiatric disorders (Faraone et al., 2021). Decades of neurocognitive research have highlighted the mechanisms underlying ADHD (Faraone et al., 2021), guiding the development of new therapies. Music therapy, the professional use of music to optimize quality of life, may have a unique position in helping individuals with ADHD by targeting brain pathways that frontline interventions (e.g., stimulant medications and cognitive Behavioral therapy) do not effectively reach (Martin-Moratinos et al., 2023).

This narrative review contributes to the growing interest in music therapy for ADHD by examining its potential neurocognitive mechanisms. While previous studies have systematically reviewed the effects of music interventions on ADHD (e.g., Martin-Moratinos et al., 2023) or narrowly focused on specific neurocognitive mechanisms (Slater and Tate, 2018; Nigg et al., 2024), this review adopts a broader perspective. By mapping multiple possible mechanisms, it aims to inform the development of more mechanistic interventions in this emerging area.

The review is organized around the following considerations. First, it focuses on specific neurocognitive mechanisms through which music therapy may benefit individuals with ADHD. These mechanisms are all supported by a general mechanism: activity-dependent neuroplasticity, the ability of the brain to reorganize its structure, function, and connections in response to intrinsic or extrinsic stimuli (Ganguly and Poo, 2013). This brain property explains why music interventions address brain disorders (Sihvonen et al., 2017). Secondly, we discuss mechanisms based both on direct evidence (studies of music therapy in ADHD) and indirect evidence (studies of music therapy in other populations). Third, we adopt the broad definition of music therapy, distinguishing between active (or creative) therapy, passive (or receptive) therapy, and mixed therapy. Active therapy involves participation in music-making, such as musical improvisation with a therapist. Passive therapy involves listening to existing music. Mixed therapy combines elements of both.

## 2 Possible mechanisms

### 2.1 Executive function enhancement

A hallmark of neurocognitive deficits in ADHD lies in executive functions (EF), an umbrella term encompassing inhibitory control, working memory, and cognitive flexibility (Diamond, 2013). Compared to individuals with typically developing peers, those with ADHD usually perform less well on EF tasks (Cortese et al., 2012; Rubia, 2018). Many prevailing theories, such as the cognitive-energetic model (Sergeant, 2005) and the dual pathway model (Sonuga-Barke, 2005), highlight the role of EF deficits in causing ADHD symptoms, positioning EF as a key target in ADHD therapies. Music interventions align with this by engaging brain regions involved in EF, such as the prefrontal cortex, through rhythm-based tasks and auditory stimuli, potentially enhancing EF and thus mitigating ADHD symptoms.

Several preliminary studies suggest that music interventions can improve EF, particularly inhibitory control, in individuals with ADHD. Rickson (2006) found that active music interventions (instructional and improvisational activities, 30–45 min per session, 1 session per week for 8 weeks) significantly improved inhibitory control and ADHD symptoms in adolescents with ADHD. Jamey et al. (2024) reported improved inhibitory control in children with ADHD through an active music intervention (gamified rhythmic training, 30 min per session, 5 sessions per week, 2 weeks). Similarly, Dursun et al. (2021) reported improving inhibitory control in adolescents with ADHD using a passive music intervention (listening to filtered Mozart music and relaxing Turkish chants, 2 h per session, 5 sessions per week, 6 weeks). However, these promising findings are limited by experimental design: the first two studies used small and unblinded samples prone to bias, and the third was a case report lacking causal rigor. These limitations highlight the need for well-controlled trials to validate the efficacy of music interventions in improving EF in ADHD.

Further evidence of the beneficial effects of music interventions on EF is provided by behavioral and neuroimaging studies derived from other populations. EF was improved in typically developing children after active therapy, such as vocal and instrumental-based musical improvisation training (Bugos et al., 2022) and rhythmic movement-based activities (Bentley et al., 2023), as well as passive therapy, such as aural training (Frischen et al., 2019; Moreno et al., 2011), or active combined with passive therapy (Jaschke et al., 2018). The effect of music interventions on EF has also been reported in various clinical populations, including autism (Janzen and Thaut, 2018), dementia (Zhang et al., 2023a), and traumatic brain injury (Thaut et al., 2009). Neuroimaging studies also suggest that musical interventions can improve brain regional or network activities underpinning EF (Colombo et al., 2020; Koshimori and Thaut, 2019; Loui, 2020). It should be noted that music interventions appear to affect the subcomponents of EF in different ways. Among children, music interventions have a particularly profound impact on inhibitory control (Rodriguez-Gomez and Talero-Gutiérrez, 2022) which is a hallmark of ADHD (Sergeant, 2005; Sonuga-Barke, 2005). In contrast, among adults, music interventions appear more impactful on “hot” EF (Frischen et al., 2022). “Hot” EF refers to executive function performance in contexts influenced by emotions, rewards, or motivation, such as self-control during delayed gratification or social interactions (Salehinejad et al., 2021). In contrast, cold EF involves performance in neutral, emotion-free contexts, with hot EF primarily linked to the medial/orbital prefrontal cortex and cold EF to the lateral prefrontal cortex (Salehinejad et al., 2021).

### 2.2 Timing improvement

An alternative explanation for ADHD symptoms is the timing deficit hypothesis (Sonuga-Barke, 2005). The deficit manifests in multiple domains, including motor timing, perception timing, and temporal foresight, affecting tasks requiring synchronization, delay discounting, and duration discrimination, resulting in ADHD symptoms (Noreika et al., 2013). Meanwhile, timing is fundamental in music activities for controlling sound duration, coordinating rhythms, and conveying expressive content. Its broad involvement enables music interventions to provide an unparalleled opportunity to exercise timing deficits in ADHD.

The effect of music interventions on timing is supported by both direct and indirect evidence. One proof-of-concept study reported that rhythm-based active interventions improved motor and perceptual timing in children with ADHD, where longer training sessions were associated with greater improvements in timing abilities (Jamey et al., 2024). Indirect evidence comes from studies on movement disorders. A narrative review highlights that passive music interventions (e.g., listening to personalized music or rhythmic auditory cues) appear to enhance perceptual timing by increasing sensitivity to external physical cues, active interventions (e.g., engaging in rhythmic movement) appear to enhance motor timing through improved motor-perceptual coordination (Devlin et al., 2023). The increased behavioral performance may be attributed to various changes in the brain, including larger activation of perceptual and motor brain regions, increased connectivity between perceptual and motor regions, and greater alignment between internal brain rhythm and external rhythmic cues (Devlin et al., 2023). Molecularly, music interventions may affect timing by affecting dopamine. In general, dopamine serves as a reward prediction error signal that regulates the neural circuits of timing (Paton and Buonomano, 2018). Music is often characterized by predictable patterns (e.g., repetitive melodies), as well as surprises (e.g., sudden tempo changes), all of which boost dopamine release (Gold et al., 2019). It should be noted that previous studies mainly examined the role of music interventions in perceptual and motor timing. Future studies may examine whether music interventions can improve motivational timing in ADHD, given the role of dopamine in motivationally related timing (e.g., delayed gratification).

### 2.3 Arousal regulation

The above two mechanisms address the task-related deficits associated with ADHD. Several models propose that ADHD symptoms are due to a non-task-related deficit: brain underarousal. According to the optimal stimulation model, ADHD is characterized by a lower arousal state, leading individuals to seek additional stimulation to compensate for the lower arousal (Zentall and Zentall, 1983). ADHD symptoms can therefore be understood as stimulus-seeking behavior driven by lower brain arousal (Strauß et al., 2018; Zhang et al., 2018, 2019). A similar explanation is found in the cognitive-energetic model (Sergeant, 2005), which proposes that low arousal levels in ADHD disrupt the brain’s ability to allocate mental resources effectively, causing difficulties with attention and executive function. These models form the basis for music interventions to manage ADHD symptoms. Music, with its rhythmic and engaging nature, provides a structured form of stimulation that enhances arousal, achieving a more optimal arousal state and improving ADHD symptoms.

Abikoff et al. (1996) and Pelham et al. (2011) reported that passively listening to music (e.g., playing background music during learning activities) helped children with ADHD focus on monotonous tasks (e.g., homework). These results can be explained by increased arousal based on the optimal stimulation model. Monotonous tasks are less stimulating environments, leading children with ADHD to seek additional stimulation to compensate for their low-level arousal, thus limiting their ability to behave properly in these tasks. In contrast, when monotonous tasks are accompanied by music, the auditory stimuli increase their arousal, reducing the need to seek additional stimulation to compensate for the lower arousal, resulting in better task focus. Notably, the optimal stimulation model can also predict that passive listening is not always beneficial - music may be detrimental to ADHD in engaging tasks in which ADHD does not need additional stimulation. However, this prediction has not been examined. Similar to the behavioral findings, a case study using EEG demonstrated that listening to classical music shifted the brain into a more alert state, suggesting that the music intervention increased arousal (Kiran, 2020).

Research from other populations supports the effect of music interventions on modulating arousal through both the autonomic nervous system (ANS) and the central nervous system (CNS). In Parkinson’s patients, a passive music intervention (Neurologic Music Therapy, 3 times per week for 5 weeks) increased the arousal of the primary motor and auditory cortexes and their coupling (Buard et al., 2019). Similarly, in patients with minimally conscious states, a passive music intervention (listening to live, previously preferred music, 5 times per week for 4 weeks) induced changes in the ascending reticular activation system, indicating higher CNS arousal (Xiao et al., 2023). This study also found increased ANS arousal, which aligns with a study involving a passive music intervention (a single session of listening to clinician-selected rhythmic musical excerpts) in a healthy population (McPherson et al., 2019). It should be noted that McPherson et al. (2019) also used an active music intervention (a single session of improvisational expressive music making), which in contrast reduced ANS arousal, suggesting different impacts of music interventions on ANS arousal. Another study examining the effect of a mixed music intervention (singing and body percussion, and rhythmic music listening within one session, 1 session per week for 6 weeks) on arousal through questionnaires found that patients with substance use disorder and post-traumatic stress disorder reported increased arousal (Hakvoort et al., 2020).

### 2.4 Default mode network modulation

Deficits in the default mode network (DMN) represent another non-task-related deficit in ADHD. Most metabolic energy in the brain is expended on spontaneous activity. This activity follows an identifiable pattern involving a large-scale brain network, including the medial prefrontal cortex, posterior cingulate cortex, and parietal cortex (Raichle, 2015). While DMN is typically involved in self-directed thinking during rest, its proper deactivation is crucial for goal-directed performance (Raichle, 2015). ADHD often exhibits abnormal DMN activity, such as weakened connectivity within the DMN and failure to deactivate it during tasks, leading to difficulties in maintaining task engagement and regulating attention (Sutcubasi et al., 2020). Thus, interventions that address DMN deficits might benefit ADHD.

Neuroimaging studies highlight the role of music in modulating DMN activity. Taruffi et al. (2017) reported that sad music enhanced self-referential thought processes by strengthening DMN connectivity. Gould Van Praag et al. (2017) found that listening to naturalistic music shifted DMN activation patterns, promoting stress reduction. Similarly, Alluri et al. (2012) showed that naturalistic music dynamically engaged DMN regions through its rhythmic and tonal features. Furthermore, Belden et al. (2020) demonstrated that classical musicians exhibited enhanced DMN connectivity, reflecting long-term neuroplastic adaptations from music training.

Although there has been no research on ADHD, several studies have demonstrated that music interventions can improve DMN in other clinical populations. Mixed interventions (e.g., frequency discrimination plus music listening) have been shown to increase DMN activity, particularly in the posterior cingulate cortex, to improve tinnitus symptoms (Krick et al., 2017) and decrease hyperconnected DMN activity within sensory regions in individuals with traumatic brain injury (Martínez-Molina et al., 2021). Studies only involving a passive music intervention also reported increased DMN activity in the posterior cingulate cortex in individuals with fibromyalgia pain (Usui et al., 2020) and schizophrenia (Yao et al., 2020). This pattern of results raises questions about whether the effects observed in mixed music interventions can be attributed to the passive intervention component. There has been no research examining the influence of active music interventions alone on DMN. Moreover, one study found that individualized music selection can enhance the effect of music interventions on a range of brain connectivity (Wu et al., 2019). Future research should investigate whether this applies to those ADHD.

### 2.5 Neural entrainment

Atypical brain oscillations have been associated with ADHD symptoms and cognitive deficits (e.g., Zhang et al., 2017a, 2017b). Addressing these atypical brain oscillations has emerged as a promising approach for developing alternative ADHD treatments (Holtmann et al., 2014). Neural entrainment, the brain’s ability to synchronize its neural oscillations with the rhythmic patterns of external stimuli, provides a powerful mechanism for targeting these irregularities (Thaut et al., 2015). Music interventions can leverage this phenomenon by using rhythmic elements to elicit brain oscillations that support desired cognitive functions (Thaut et al., 2015; Vuilleumier and Trost, 2015). For example, individuals with ADHD often exhibit reduced fast oscillations, which are closely linked to deficits in cognitive functioning (Zhang et al., 2017a, 2017b). Music interventions can employ rhythms that match the frequency of these fast oscillations, enhancing them through neural entrainment and thereby improving cognitive functioning.

The evidence supporting neural entrainment comes primarily from passive music interventions conducted in non-ADHD populations. An MEG study in a normal population found that when musical rhythms were played at frequencies below 8 Hz, brain oscillations also displayed that specific frequency and its harmonics for perceiving and decoding musical units (Doelling and Poeppel, 2015). Besides influencing music processing, this study also found that neural entrainment affected general cognitive processes based on the same brain oscillations. A second line of evidence comes from neurological disorders. Researchers used a specific passive music intervention – Neurologic Music Therapy – to elicit desired brain oscillations via neural entrainment (Thaut et al., 2015; Braun Janzen et al., 2022).

### 2.6 Affective management

Affective states are also difficult to manage for ADHD (Mulraney et al., 2016; Sandstrom et al., 2021). Affect consists of emotions and moods; emotions are intense, short-lived emotional responses to specific events or stimuli, while moods are diffuse, longer-lasting emotional reactions that are not necessarily tied to a particular event or stimulus. Both affective states are often dysregulated in ADHD. Emotional dysregulation is frequently observed throughout the lifetime of those with ADHD, significantly contributing to its psychosocial impairments (Shaw et al., 2014). Mood disorders are separate diagnoses in the DSM-5 [American Psychiatric Association (APA), 2013]. Those with ADHD are predisposed to mood disorders, such as disruptive mood dysregulation disorder (Mulraney et al., 2016) and bipolar disorder (Sandstrom et al., 2021). Drug therapies appear less effective at addressing affective dysregulation in ADHD (Lenzi et al., 2018), highlighting the need for alternative interventions to address affective difficulties. Music has long been known to moderate affective states and may serve as a unique stimulus to address affective dysregulation in ADHD.

Several systematic reviews have discussed how music interventions impact affective regulation in populations other than ADHD (e.g., Gold et al., 2009; Koelsch, 2014; Moore, 2013), and interested readers can refer to these sources for broader findings. Here, we focus specifically on the evidence related to ADHD. A study on adults with ADHD demonstrated that one-session listening to Mozart’s music led to a decrease in negative mood (Zimmermann et al., 2019). The positive effect of a passive music intervention (listening to relaxing music, 15 min before and 30 min during each CBT session, 12 weekly sessions) on improving emotion was also found in a preliminary case report (Zemestani et al., 2023). Another study provided more nuanced insights into the effects on affective regulation in ADHD, highlighting that a mixed music intervention (improvisation and passive receptive listening to music, 50 min per session, 2 sessions per week, 12 weeks). alter neurophysiological processes relevant to affective regulation, such as 5-HT, cortisol, blood pressure, and heart rate (Park et al., 2023). Given these promising results, future research may explore how different types of music interventions (e.g., active vs. passive) impact specific aspects of affective regulation (e.g., emotional reactivity and mood stabilization) and their lasting impact on psychosocial functioning.

### 2.7 Social bonding

The inherent synchrony of music has historically served as a coalition signaling system that fosters social cohesion and trust among individuals - the social bonding theory of music (Savage et al., 2021). Meanwhile, social bonding poses another challenge for those with ADHD. Deficient social cognition involving prosody perception and the theory of mind is often observed in ADHD (Bora and Pantelis, 2016; Uekermann et al., 2010), leading to poor prosocial skills, aggressive conduct problems, and compromised quality of life (Arango-Tobón et al., 2023; Hay et al., 2010; Velő et al., 2021). Drawing on the social bonding perspective of music, it is plausible that music interventions could serve as an effective medium for cooperative learning, enabling individuals to align their intentions with group actions and potentially mitigating social impairments in ADHD.

A preliminary study using a pre-post design found that an active music intervention (group-based music movement to music and improvisation, 50 min per session, 1 session per week, 5 weeks) improved a range of social skills in ADHD (Gooding, 2011). Research from other populations lends further support to the effect of music interventions on social bonding. A recent review explored the influence of passive music interventions on prosocial behavior in typically developing populations (Grimani et al., 2024). This synthesis suggests that carefully designed music interventions, for example, focusing on factors like lyrics and the emotional effects of the music, can effectively promote prosocial behavior (e.g., donating money and volunteering). The second line of evidence comes from substantial autism research. It has been shown that active music interventions can enhance social bonding through multiple mechanisms, such as increasing social engagement (Thompson et al., 2014), increasing joint attention (LaGasse, 2014), improving social skills (Mössler et al., 2019), and fostering theory of mind (Silarat, 2022).

## 3 Discussion

This narrative review summarizes seven mechanisms by which music interventions can address neurocognitive deficits in ADHD, based on emerging studies and substantial research from other populations. Multiple components are often involved in music interventions. For example, an intervention could include both active and passive components, with each component further comprising multiple elements. As a result of this complexity, it might be possible for music interventions to simultaneously influence ADHD via multiple mechanisms (e.g., Jamey et al., 2024). On the other hand, the complexity also calls for future studies to isolate the causal effects of specific music components on ADHD.

Future studies should further tailor music interventions based on ADHD characteristics. Previous studies have used general music interventions to treat ADHD. In the meantime, research shows that certain rhythmic stimuli, such as white noise (Nigg et al., 2024) and binaural beats (Basu and Banerjee, 2023) can help to reduce ADHD symptoms and improve cognitive functioning. The rhythmic stimuli alone are not considered music, but they may be carefully incorporated into music interventions to enhance their effectiveness. Another approach to tailoring music interventions for ADHD is to prescribe interventions based on individual deficit profiles. The review revealed that music interventions may address several neurocognitive deficits. It should be noted that these neurocognitive deficits were mainly identified through group-level analyses. It is typical of ADHD to display heterogeneity in these deficits (Faraone et al., 2021). As a result of this heterogeneity, those with ADHD may respond differently to different treatments (e.g., non-pharmacological interventions; Zhang, 2023; Zhang et al., 2023b). Thus, music interventions may be tailored based on individual deficit profiles to achieve larger effects.

Overall, music interventions for ADHD are still in their infancy. In this review, we discuss potential mechanisms for studying the effects of music interventions on ADHD in the future. A more tailored approach to music interventions is necessary given the nature of neurocognitive deficits in ADHD.

## Data Availability

The original contributions presented in the study are included in the article/Supplementary material, further inquiries can be directed to the corresponding authors.

## References

[ref1] Abikoff H. Courtney M. E. Szeibel P. J. Koplewicz H. S. (1996). The effects of auditory stimulation on the arithmetic performance of children with ADHD and nondisabled children. J. Learn. Disabil. 29, 238–246. doi: 10.1177/002221949602900302, PMID: 8732885

[ref2] Alluri V. Toiviainen P. Jääskeläinen I. P. Glerean E. Sams M. Brattico E. (2012). Large-scale brain networks emerge from dynamic processing of musical timbre, key and rhythm. NeuroImage 59, 3677–3689. doi: 10.1016/j.neuroimage.2011.11.019, PMID: 22116038

[ref3] American Psychiatric Association (APA) (2013). Diagnostic and statistical manual of mental disorders: DSM-5. 5th Edn. Washington DC: American Psychiatric Association.

[ref4] Arango-Tobón O. E. Guevara Solórzano A. Orejarena Serrano S. J. Olivera-La Rosa A. (2023). Social cognition and prosocial behavior in children with attention deficit hyperactivity disorder: A systematic review. Healthcare 11:1366. doi: 10.3390/healthcare11101366, PMID: 37239652 PMC10218260

[ref5] Basu S. Banerjee B. (2023). Potential of binaural beats intervention for improving memory and attention: insights from meta-analysis and systematic review. Psychol. Res. 87, 951–963. doi: 10.1007/s00426-022-01706-7, PMID: 35842538

[ref6] Belden A. Zeng T. Przysinda E. Anteraper S. A. Whitfield-Gabrieli S. Loui P. (2020). Improvising at rest: differentiating jazz and classical music training with resting state functional connectivity. NeuroImage 207:116384. doi: 10.1016/j.neuroimage.2019.116384, PMID: 31760149

[ref7] Bentley L. A. Eager R. Savage S. Nielson C. White S. L. J. Williams K. E. (2023). A translational application of music for preschool cognitive development: RCT evidence for improved executive function, self-regulation, and school readiness. Dev. Sci. 26:e13358. doi: 10.1111/desc.13358, PMID: 36511452

[ref8] Bora E. Pantelis C. (2016). Meta-analysis of social cognition in attention-deficit/hyperactivity disorder (ADHD): comparison with healthy controls and autistic spectrum disorder. Psychol. Med. 46, 699–716. doi: 10.1017/S0033291715002573, PMID: 26707895

[ref9] Braun Janzen T. Koshimori Y. Richard N. M. Thaut M. H. (2022). Rhythm and music-based interventions in motor rehabilitation: current evidence and future perspectives. Front. Hum. Neurosci. 15:789467. doi: 10.3389/fnhum.2021.789467, PMID: 35111007 PMC8801707

[ref10] Buard I. Dewispelaere W. B. Thaut M. Kluger B. M. (2019). Preliminary neurophysiological evidence of altered cortical activity and connectivity with neurologic music therapy in Parkinson’s disease. Front. Neurosci. 13:105. doi: 10.3389/fnins.2019.00105 30837830 PMC6390231

[ref11] Bugos J. A. DeMarie D. Stokes C. Power P. (2022). Multimodal music training enhances executive functions in children: results of a randomized controlled trial. Ann. N. Y. Acad. Sci. 1516, 95–105. doi: 10.1111/nyas.14857, PMID: 35899371

[ref12] Chanda M. L. Levitin D. J. (2013). The neurochemistry of music. Trends Cogn. Sci. 17, 179–193. doi: 10.1016/j.tics.2013.02.007, PMID: 23541122

[ref13] Colombo P. J. Habibi A. Alain C. (2020). Editorial: music training, neural plasticity, and executive function. Front. Integr. Neurosci. 14:41. doi: 10.3389/fnint.2020.00041 32903753 PMC7438867

[ref14] Cortese S. Kelly C. Chabernaud C. Proal E. Di Martino A. Milham M. P.. (2012). Toward systems neuroscience of ADHD: A meta-analysis of 55 fMRI studies. Am. J. Psychiatry 169, 1038–1055. doi: 10.1176/appi.ajp.2012.11101521, PMID: 22983386 PMC3879048

[ref15] Devlin K. Kang K. Pantelyat A. (2023). Music therapy and music-based interventions in neurology: Perspectives on research and practice. New Jersey: Humana Press.

[ref16] Diamond A. (2013). Executive functions. Annu. Rev. Psychol. 64, 135–168. doi: 10.1146/annurev-psych-113011-143750, PMID: 23020641 PMC4084861

[ref17] Doelling K. B. Poeppel D. (2015). Cortical entrainment to music and its modulation by expertise. Proc. Natl. Acad. Sci. 112, E6233–E6242. doi: 10.1073/pnas.1508431112, PMID: 26504238 PMC4653203

[ref18] Dursun P. Fidan U. Karayagiz S. (2021). Probable role of listening therapy in the management of ADHD symptoms: three case studies. Curr. Psychol. 40, 4219–4234. doi: 10.1007/s12144-021-01419-x

[ref19] Faraone S. V. Banaschewski T. Coghill D. Zheng Y. Biederman J. Bellgrove M. A.. (2021). The world federation of ADHD international consensus statement: 208 evidence-based conclusions about the disorder. Neurosci. Biobehav. Rev. 128, 789–818. doi: 10.1016/j.neubiorev.2021.01.022, PMID: 33549739 PMC8328933

[ref20] Frischen U. Schwarzer G. Degé F. (2019). Comparing the effects of rhythm-based music training and pitch-based music training on executive functions in preschoolers. Front. Integr. Neurosci. 13:41. doi: 10.3389/fnint.2019.00041 31507385 PMC6718722

[ref21] Frischen U. Schwarzer G. Degé F. (2022). Music training and executive functions in adults and children: what role do hot executive functions play? Z. Erzieh. 25, 551–578. doi: 10.1007/s11618-022-01103-1

[ref22] Ganguly K. Poo M. (2013). Activity-dependent neural plasticity from bench to bedside. Neuron 80, 729–741. doi: 10.1016/j.neuron.2013.10.028, PMID: 24183023

[ref23] Gold B. P. Pearce M. T. Mas-Herrero E. Dagher A. Zatorre R. J. (2019). Predictability and uncertainty in the pleasure of music: A reward for learning? J. Neurosci. 39, 9397–9409. doi: 10.1523/JNEUROSCI.0428-19.2019, PMID: 31636112 PMC6867811

[ref24] Gold C. Solli H. P. Krüger V. Lie S. A. (2009). Dose–response relationship in music therapy for people with serious mental disorders: systematic review and meta-analysis. Clin. Psychol. Rev. 29, 193–207. doi: 10.1016/j.cpr.2009.01.001, PMID: 19269725

[ref25] Gooding L. F. (2011). The effect of a music therapy social skills training program on improving social competence in children and adolescents with social skills deficits. J. Music. Ther. 48, 440–462. doi: 10.1093/jmt/48.4.440, PMID: 22506299

[ref26] Gould Van Praag C. D. Garfinkel S. N. Sparasci O. Mees A. Philippides A. O. Ware M.. (2017). Mind-wandering and alterations to default mode network connectivity when listening to naturalistic versus artificial sounds. Sci. Rep. 7:45273. doi: 10.1038/srep45273 28345604 PMC5366899

[ref27] Grimani A. Moog A. Vlaev I. (2024). Analysis of music-exposure interventions for impacting prosocial behaviour via behaviour change techniques and mechanisms of action: A rapid review. Curr. Psychol. 43, 1136–1168. doi: 10.1007/s12144-023-04406-6

[ref28] Hakvoort L. De Jong S. Van De Ree M. Kok T. Macfarlane C. De Haan H. (2020). Music therapy to regulate arousal and attention in patients with substance use disorder and posttraumatic stress disorder: A feasibility study. J. Music. Ther. 57, 353–378. doi: 10.1093/jmt/thaa007, PMID: 32651585

[ref29] Hay D. F. Hudson K. Liang W. (2010). Links between preschool children’s prosocial skills and aggressive conduct problems: the contribution of ADHD symptoms. Early Child. Res. Q. 25, 493–501. doi: 10.1016/j.ecresq.2010.01.003

[ref30] Holtmann M. Sonuga-Barke E. Cortese S. Brandeis D. (2014). Neurofeedback for ADHD. Child Adolesc. Psychiatr. Clin. N. Am. 23, 789–806. doi: 10.1016/j.chc.2014.05.006, PMID: 25220087

[ref31] Jamey K. Laflamme H. Foster N. E. V. Rigoulot S. Kotz S. A. Bella S. D. (2024). Can you beat the music? Validation of a Gamified Rhythmic Training in Children with ADHD. Psychiatry Clinic. Psychol. doi: 10.1101/2024.03.19.24304539 (preprint). 41053410

[ref32] Janzen T. B. Thaut M. H. (2018). Rethinking the role of music in the neurodevelopment of autism spectrum disorder. Music Sci. 1:205920431876963. doi: 10.1177/2059204318769639

[ref33] Jaschke A. C. Honing H. Scherder E. J. A. (2018). Longitudinal analysis of music education on executive functions in primary school children. Front. Neurosci. 12:103. doi: 10.3389/fnins.2018.00103 29541017 PMC5835523

[ref34] Kiran F. (2020). *Exploring effects of background music in A serious game on attention by means of EEG signals in children* [master of science in computer science, Louisiana State University and agricultural and mechanical college]. Available at: https://repository.lsu.edu/gradschool_theses/5151/

[ref35] Koelsch S. (2014). Brain correlates of music-evoked emotions. Nat. Rev. Neurosci. 15, 170–180. doi: 10.1038/nrn3666, PMID: 24552785

[ref36] Koshimori Y. Thaut M. H. (2019). New perspectives on music in rehabilitation of executive and attention functions. Front. Neurosci. 13:1245. doi: 10.3389/fnins.2019.01245 31803013 PMC6877665

[ref37] Krick C. M. Argstatter H. Grapp M. Plinkert P. K. Reith W. (2017). Heidelberg neuro-music therapy enhances task-negative activity in tinnitus patients. Front. Neurosci. 11:384. doi: 10.3389/fnins.2017.00384 28736515 PMC5500649

[ref38] LaGasse A. B. (2014). Effects of a music therapy group intervention on enhancing social skills in children with autism. J. Music. Ther. 51, 250–275. doi: 10.1093/jmt/thu012, PMID: 25053766

[ref39] Lenzi F. Cortese S. Harris J. Masi G. (2018). Pharmacotherapy of emotional dysregulation in adults with ADHD: A systematic review and meta-analysis. Neurosci. Biobehav. Rev. 84, 359–367. doi: 10.1016/j.neubiorev.2017.08.010, PMID: 28837827

[ref40] Loui P. (2020). Neuroscientific insights for improved outcomes in music-based interventions. Music Sci. 3:205920432096506. doi: 10.1177/2059204320965065

[ref41] Martínez-Molina N. Siponkoski S.-T. Kuusela L. Laitinen S. Holma M. Ahlfors M.. (2021). Resting-state network plasticity induced by music therapy after traumatic brain injury. Neural Plast. 2021, 1–18. doi: 10.1155/2021/6682471, PMID: 33763126 PMC7964116

[ref42] Martin-Moratinos M. Bella-Fernández M. Blasco-Fontecilla H. (2023). Effects of music on attention-deficit/hyperactivity disorder (ADHD) and potential application in serious video games: systematic review. J. Med. Internet Res. 25:e37742. doi: 10.2196/37742, PMID: 37171837 PMC10221503

[ref43] McPherson T. Berger D. Alagapan S. Fröhlich F. (2019). Active and passive rhythmic music therapy interventions differentially modulate sympathetic autonomic nervous system activity. J. Music. Ther. 56, 240–264. doi: 10.1093/jmt/thz007, PMID: 31175814 PMC6693240

[ref44] Moore K. S. (2013). A systematic review on the neural effects of music on emotion regulation: implications for music therapy practice. J. Music. Ther. 50, 198–242. doi: 10.1093/jmt/50.3.198, PMID: 24568004

[ref45] Moreno S. Bialystok E. Barac R. Schellenberg E. G. Cepeda N. J. Chau T. (2011). Short-term music training enhances verbal intelligence and executive function. Psychol. Sci. 22, 1425–1433. doi: 10.1177/0956797611416999, PMID: 21969312 PMC3449320

[ref46] Mössler K. Gold C. Aßmus J. Schumacher K. Calvet C. Reimer S.. (2019). The therapeutic relationship as predictor of change in music therapy with young children with autism Spectrum disorder. J. Autism Dev. Disord. 49, 2795–2809. doi: 10.1007/s10803-017-3306-y, PMID: 28936692

[ref47] Mulraney M. Schilpzand E. J. Hazell P. Nicholson J. M. Anderson V. Efron D.. (2016). Comorbidity and correlates of disruptive mood dysregulation disorder in 6–8-year-old children with ADHD. Eur. Child Adolesc. Psychiatry 25, 321–330. doi: 10.1007/s00787-015-0738-9, PMID: 26122202

[ref48] Nigg J. T. Bruton A. Kozlowski M. B. Johnstone J. M. Karalunas S. L. (2024). Systematic review and meta-analysis: do White noise or pink noise help with task performance in youth with attention-deficit/hyperactivity disorder or with elevated attention problems? J. Am. Acad. Child Adolesc. Psychiatry S0890856724000741, 778–788. doi: 10.1016/j.jaac.2023.12.014 PMC11283987 38428577

[ref49] Noreika V. Falter C. M. Rubia K. (2013). Timing deficits in attention-deficit/hyperactivity disorder (ADHD): evidence from neurocognitive and neuroimaging studies. Neuropsychologia 51, 235–266. doi: 10.1016/j.neuropsychologia.2012.09.036, PMID: 23022430

[ref50] Park J.-I. Lee I.-H. Lee S.-J. Kwon R.-W. Choo E.-A. Nam H.-W.. (2023). Effects of music therapy as an alternative treatment on depression in children and adolescents with ADHD by activating serotonin and improving stress coping ability. BMC Comp Med Therap. 23:73. doi: 10.1186/s12906-022-03832-6 PMC9987133 36879223

[ref51] Paton J. J. Buonomano D. V. (2018). The neural basis of timing: distributed mechanisms for diverse functions. Neuron 98, 687–705. doi: 10.1016/j.neuron.2018.03.045, PMID: 29772201 PMC5962026

[ref52] Pelham W. E. Waschbusch D. A. Hoza B. Gnagy E. M. Greiner A. R. Sams S. E.. (2011). Music and video as distractors for boys with ADHD in the classroom: comparison with controls, individual differences, and medication effects. J. Abnorm. Child Psychol. 39, 1085–1098. doi: 10.1007/s10802-011-9529-z, PMID: 21695447

[ref53] Raichle M. E. (2015). The Brain’s default mode network. Annu. Rev. Neurosci. 38, 433–447. doi: 10.1146/annurev-neuro-071013-014030, PMID: 25938726

[ref54] Rickson D. J. (2006). Instructional and improvisational models of music therapy with adolescents who have attention deficit hyperactivity disorder (ADHD): A comparison of the effects on motor impulsivity. J. Music. Ther. 43, 39–62. doi: 10.1093/jmt/43.1.39, PMID: 16671837

[ref55] Rodriguez-Gomez D. A. Talero-Gutiérrez C. (2022). Effects of music training in executive function performance in children: A systematic review. Front. Psychol. 13:968144. doi: 10.3389/fpsyg.2022.968144, PMID: 36003104 PMC9393548

[ref56] Rubia K. (2018). Cognitive neuroscience of attention deficit hyperactivity disorder (ADHD) and its clinical translation. Front. Hum. Neurosci. 12:100. doi: 10.3389/fnhum.2018.00100 29651240 PMC5884954

[ref9001] Salehinejad M. A. Ghanavati E. Rashid M. H. A. Nitsche M. A. (2021). Hot and cold executive functions in the brain: A prefrontal-cingular network. Brain Neurosci. Adv. 5:23982128211007769. doi: 10.1177/23982128211007769 33997292 PMC8076773

[ref57] Sandstrom A. Perroud N. Alda M. Uher R. Pavlova B. (2021). Prevalence of attention-deficit/hyperactivity disorder in people with mood disorders: A systematic review and meta-analysis. Acta Psychiatr. Scand. 143, 380–391. doi: 10.1111/acps.13283, PMID: 33528847

[ref58] Savage P. E. Loui P. Tarr B. Schachner A. Glowacki L. Mithen S.. (2021). Music as a coevolved system for social bonding. Behav. Brain Sci. 44:e59. doi: 10.1017/S0140525X20000333, PMID: 32814608

[ref59] Sergeant J. A. (2005). Modeling attention-deficit/hyperactivity disorder: A critical appraisal of the cognitive-energetic model. Biol. Psychiatry 57, 1248–1255. doi: 10.1016/j.biopsych.2004.09.010, PMID: 15949995

[ref60] Shaw P. Stringaris A. Nigg J. Leibenluft E. (2014). Emotion dysregulation in attention deficit hyperactivity disorder. Am. J. Psychiatry 171, 276–293. doi: 10.1176/appi.ajp.2013.13070966, PMID: 24480998 PMC4282137

[ref61] Sihvonen A. J. Särkämö T. Leo V. Tervaniemi M. Altenmüller E. Soinila S. (2017). Music-based interventions in neurological rehabilitation. Lancet Neurol. 16, 648–660. doi: 10.1016/S1474-4422(17)30168-0, PMID: 28663005

[ref62] Silarat C. (2022). Piano lessons: fostering theory of mind in ASD through imitation. Int. J. Disabil. Dev. Educ. 69, 154–169. doi: 10.1080/1034912X.2021.1947473

[ref63] Slater J. L. Tate M. C. (2018). Timing deficits in ADHD: insights from the neuroscience of musical rhythm. Front. Comput. Neurosci. 12:51. doi: 10.3389/fncom.2018.00051 30034331 PMC6043674

[ref64] Sonuga-Barke E. J. S. (2005). Causal models of attention-deficit/hyperactivity disorder: from common simple deficits to multiple developmental pathways. Biol. Psychiatry 57, 1231–1238. doi: 10.1016/j.biopsych.2004.09.008, PMID: 15949993

[ref65] Strauß M. Ulke C. Paucke M. Huang J. Mauche N. Sander C.. (2018). Brain arousal regulation in adults with attention-deficit/hyperactivity disorder (ADHD). Psychiatry Res. 261, 102–108. doi: 10.1016/j.psychres.2017.12.043, PMID: 29291475

[ref66] Sutcubasi B. Metin B. Kurban M. K. Metin Z. E. Beser B. Sonuga-Barke E. (2020). Resting-state network dysconnectivity in ADHD: A system-neuroscience-based meta-analysis. World J. Biol. Psychiatry 21, 662–672. doi: 10.1080/15622975.2020.1775889, PMID: 32468880

[ref67] Taruffi L. Pehrs C. Skouras S. Koelsch S. (2017). Effects of sad and happy music on mind-wandering and the default mode network. Sci. Rep. 7:14396. doi: 10.1038/s41598-017-14849-0 29089542 PMC5663956

[ref68] Thaut M. H. Gardiner J. C. Holmberg D. Horwitz J. Kent L. Andrews G.. (2009). Neurologic music therapy improves executive function and emotional adjustment in traumatic brain injury rehabilitation. Ann. N. Y. Acad. Sci. 1169, 406–416. doi: 10.1111/j.1749-6632.2009.04585.x, PMID: 19673815

[ref69] Thaut M. H. McIntosh G. C. Hoemberg V. (2015). Neurobiological foundations of neurologic music therapy: rhythmic entrainment and the motor system. Front. Psychol. 5:1185. doi: 10.3389/fpsyg.2014.01185, PMID: 25774137 PMC4344110

[ref70] Thompson G. A. McFerran K. S. Gold C. (2014). Family-centred music therapy to promote social engagement in young children with severe autism spectrum disorder: A randomized controlled study. Child Care Health Dev. 40, 840–852. doi: 10.1111/cch.12121, PMID: 24261547

[ref71] Tramo M. J. (2001). Music of the hemispheres. Science 291, 54–56. doi: 10.1126/science.10.1126/SCIENCE.1056899, PMID: 11192009

[ref72] Uekermann J. Kraemer M. Abdel-Hamid M. Schimmelmann B. G. Hebebrand J. Daum I.. (2010). Social cognition in attention-deficit hyperactivity disorder (ADHD). Neurosci. Biobehav. Rev. 34, 734–743. doi: 10.1016/j.neubiorev.2009.10.009, PMID: 19857516

[ref73] Usui C. Kirino E. Tanaka S. Inami R. Nishioka K. Hatta K.. (2020). Music intervention reduces persistent fibromyalgia pain and alters functional connectivity between the insula and default mode network. Pain Med. 21, 1546–1552. doi: 10.1093/pm/pnaa071, PMID: 32330259

[ref74] Velő S. Keresztény Á. Ferenczi-Dallos G. Pump L. Móra K. Balázs J. (2021). The association between prosocial behaviour and peer relationships with comorbid externalizing disorders and quality of life in treatment-Naïve children and adolescents with attention deficit hyperactivity disorder. Brain Sci. 11:475. doi: 10.3390/brainsci11040475, PMID: 33918547 PMC8069734

[ref75] Vuilleumier P. Trost W. (2015). Music and emotions: from enchantment to entrainment. Ann. N. Y. Acad. Sci. 1337, 212–222. doi: 10.1111/nyas.12676, PMID: 25773637

[ref76] Wu K. Anderson J. Townsend J. Frazier T. Brandt A. Karmonik C. (2019). Characterization of functional brain connectivity towards optimization of music selection for therapy: A fMRI study. Int. J. Neurosci. 129, 882–889. doi: 10.1080/00207454.2019.1581189, PMID: 30744538

[ref77] Xiao X. Chen W. Zhang X. (2023). The effect and mechanisms of music therapy on the autonomic nervous system and brain networks of patients of minimal conscious states: A randomized controlled trial. Front. Neurosci. 17:1182181. doi: 10.3389/fnins.2023.1182181 37250411 PMC10213399

[ref78] Yao Y. He H. Duan M. Li S. Li C. Chen X.. (2020). The effects of music intervention on pallidum-DMN circuit of schizophrenia. Biomed. Res. Int. 2020, 1–10. doi: 10.1155/2020/4107065 PMC7525302 33015164

[ref79] Yinger O. S. Gooding L. (2014). Music therapy and music medicine for children and adolescents. Child Adolesc. Psychiatr. Clin. N. Am. 23, 535–553. doi: 10.1016/j.chc.2013.03.003, PMID: 24975624

[ref80] Zemestani M. Azadbakht M. Storch E. A. (2023). Preliminary evaluation of music-based emotion-regulation skills to augment CBT for adolescents with ADHD. Music. Sci. 27, 757–779. doi: 10.1177/10298649221146050

[ref81] Zentall S. S. Zentall T. R. (1983). Optimal stimulation: A model of disordered activity and performance in normal and deviant children. Psychol. Bull. 94, 446–471. doi: 10.1037/0033-2909.94.3.446 6657825

[ref82] Zhang D.-W. (2023). Perspectives on heterogeneity-informed cognitive training for attention-deficit/hyperactivity disorder. Front. Psych. 13:1100008. doi: 10.3389/fpsyt.2022.1100008 PMC9878183 36713921

[ref83] Zhang D.-W. Johnstone S. J. Li H. Barry R. J. Clarke A. R. Zhao Q.. (2019). Time effects on resting EEG in children with/without AD/HD. Brain Topogr. 32, 286–294. doi: 10.1007/s10548-018-0690-3, PMID: 30498871

[ref84] Zhang D.-W. Johnstone S. J. Roodenrys S. Luo X. Li H. Wang E.. (2018). The role of resting-state EEG localized activation and central nervous system arousal in executive function performance in children with attention-deficit/hyperactivity disorder. Clin. Neurophysiol. 129, 1192–1200. doi: 10.1016/j.clinph.2018.03.009, PMID: 29653296

[ref85] Zhang D.-W. Johnstone S. J. Sauce B. Arns M. Sun L. Jiang H. (2023b). Remote neurocognitive interventions for attention-deficit/hyperactivity disorder – opportunities and challenges. Prog. Neuro-Psychopharmacol. Biol. Psychiatry 127:110802. doi: 10.1016/j.pnpbp.2023.110802 37257770

[ref86] Zhang D.-W. Li H. Wu Z. Zhao Q. Song Y. Liu L.. (2017b). Electroencephalogram theta/Beta ratio and spectral Power correlates of executive functions in children and adolescents with AD/HD. J. Atten. Disord. 23, 721–732. doi: 10.1177/1087054717718263 28689463

[ref87] Zhang D.-W. Roodenrys S. Li H. Barry R. J. Clarke A. R. Wu Z.. (2017a). Atypical interference control in children with AD/HD with elevated theta/beta ratio. Biol. Psychol. 128, 82–88. doi: 10.1016/j.biopsycho.2017.07.009 28734758

[ref88] Zhang J. Yu Z. Zhang N. Zhao W. Wei B. He R.. (2023a). Does music intervention relieve depression or anxiety in people living with dementia? A systematic review and meta-analysis. Aging Ment. Health 27, 1864–1875. doi: 10.1080/13607863.2023.2214091 37243671

[ref89] Zimmermann M. B. Diers K. Strunz L. Scherbaum N. Mette C. (2019). Listening to Mozart improves current mood in adult ADHD – A randomized controlled pilot study. Front. Psychol. 10:1104. doi: 10.3389/fpsyg.2019.01104 31156516 PMC6529778

