# Supplementary material for: Rhythms of relief: perspectives on neurocognitive mechanisms of music interventions in ADHD
Source: Front Psychol. 2025 Mar 3;16:1476928. doi: 10.3389/fpsyg.2025.1476928 (PMC11911488; doi:10.3389/fpsyg.2025.1476928)
Supplement: Supplementary file 1 [file Data_Sheet_1.pdf]

Table 1. Overview of Possible Neurocognitive Mechanisms of Music Interventions and Corresponding Studies in ADHD.

Possible Mechanism	Description	Evidence type	ADHD Population	Experimental Design	Music Intervention Type	Training Duration/Frequency	Outcome Measures
Executive Function Enhancement	Music interventions stimulate brain regions involved in deficient executive functions in ADHD, such as the prefrontal cortex, thus mitigating symptoms like poor inhibitory control and working memory deficits.	Direct	Rickson et al. (2006): Adolescents, 11–16 years old, mean age 13, all males.<br><br>Jamey et al. (2024): Children (n = 27, 7–13 years, 3 females).<br><br>Dursun et al. (2021): Adolescents (n = 3, aged 14, 15, and 17, 1 female, 2 males)	Rickson et al. (2006): Experimental design.<br><br>Jamey et al. (2024): Experimental design.<br><br>Dursun et al. (2021): Case report	Rickson et al. (2006): Active, instructional and improvisational activities.<br><br>Jamey et al. (2024): Active, gamified rhythmic training.<br><br>Dursun et al. (2021): Passive, listening therapy based on filtered Mozart music and relaxing Turkish chants.	Rickson et al. (2006): 30–45 minutes per session, 1 session per week, 8 weeks.<br><br>Jamey et al. (2024): 30 minutes per session, 5 sessions per week, 2 weeks.<br><br>Dursun et al. (2021): 2 hours per session, 5 sessions per week, 6 weeks.	Rickson et al. (2006): Synchronized Tapping Task (STT), Conners' Rating Scales (teacher and parent versions).<br><br>Jamey et al. (2024): Alignment Test and paced tapping tasks, set-switching, Flanker, Go/No-Go tasks.<br><br>Dursun et al. (2021): Continuous performance task, Conners' Rating Scales (parent and teacher versions), and ADHD self-report scale
Timing Improvement	Rhythmic music engages brain networks like the cerebellum and basal ganglia, addressing deficiencies in motor and perceptual timing seen in ADHD.	Direct	Jamey et al. (2024): Children (n = 27, 7–13 years, 3 females).	Jamey et al. (2024): Experimental design.	Jamey et al. (2024): Active, gamified rhythmic training.	Jamey et al. (2024): 30 minutes per session, 5 sessions per week, 2 weeks.	Jamey et al. (2024): Alignment Test and paced tapping tasks, set-switching, Flanker, Go/No-Go tasks.
Arousal Regulation	Music stimulates the autonomic nervous system	Direct	Abikoff et al. (1996): Children (n = 20, 7.5–13 years,	Abikoff et al. (1996): Experimental	Abikoff et al. (1996): Passive, individualized	Abikoff et al. (1996): 10 minutes per session, 1	Abikoff et al. (1996): Arithmetic performance.

	and modulates cortical arousal, helping to counteract ADHD-related under-arousal and improve cognitive performance.		mean age 9.9, all males).<br><br>Pelham et al. (2011): Children (n = 41, 7.7–12.6 years, mean age 9.8, all males).<br><br>Kiran (2020): Children (n = 10, 8–12 years, 3 females).	design.<br><br>Pelham et al. (2011): Experimental design<br><br>Kiran (2020): Experimental design.	background music.<br><br>Pelham et al. (2011): Passive, rock or rap based background music.<br><br>Kiran (2020): Passive, classical music.	session.<br><br>Pelham et al. (2011): 45 minutes per session, 4 sessions per week, 6 weeks.<br><br>Kiran (2020): during playing Tetris.	Pelham et al. (2011): On-task behavior, rule violations, and teacher prompts.<br><br>Kiran (2020): EEG Alpha and Beta waves.
Default Mode Network Modulation	Music regulates the Default Mode Network, reducing mind-wandering and enhancing task engagement, mitigating deficits in ADHD.	Indirect					
Neural Entrainment	Rhythmic music aligns brain oscillations with external rhythms, improving cognitive processes often disrupted in ADHD.	Indirect					

Affective Management	Music engages the limbic system to modulate emotions, reducing emotional dysregulation, impulsivity, and irritability in ADHD.	Direct	Zimmermann et al. (2019): Adults (n = 40, mean age 33 years, 20 males, 20 females).<br><br>Zemestani et al. (2023): Adolescents (n = 8, 13–17 years, mean age 15.87, 5 females, 3 males).<br><br>Park et al. (2019): Children and adolescents (n = 36, 9–15 years, mean age 12.2, 18 males, 18 females)	Zimmermann et al. (2019): Experimental design.<br><br>Zemestani et al. (2023): Case report.<br><br>Park et al. (2019): Experimental design.	Zimmermann et al. (2019): Passive, listening to Mozart's piano sonata.<br><br>Zemestani et al. (2023): Passive, individualized listening to relaxing music.<br><br>Park et al. (2019): Mixed, active (improvisation) and passive (receptive listening to music).	Zimmermann et al. (2019): 10 minutes per session, 1 session.<br><br>Zemestani et al. (2023): 15 minutes before and 30 minutes during each CBT session, 12 weekly sessions.<br><br>Park et al. (2019): 50 minutes per session, 2 sessions per week, 12 weeks.	Zimmermann et al. (2019): Current Mood Scale and Global Mood-Arousal Scale.<br><br>Zemestani et al. (2023): Conners' Parent Rating Scale-Revised and Emotion Regulation Questionnaire for Children and Adolescents.<br><br>Park et al. (2019): Serotonin levels, cortisol levels, systolic and diastolic blood pressure, heart rate, Children's Depression Inventory, and Daily Hassles Questionnaire.
Social Bonding	Music fosters synchrony and shared emotional experiences, enhancing prosocial behaviors by activating reward pathways.	Direct	Gooding (2011): Children and adolescents (n = 44, 6–17 years, gender not specified).	Gooding (2011): Quasi-experimental design.	Gooding (2011): Active, group-based music movement to music and improvisation.	Gooding (2011): 50 minutes per session, 1 session per week, 5 weeks.	Gooding (2011): Self-ratings, teacher ratings, researcher ratings of social competence, and behavioral observations of on-task behavior.
